# Disseminated Multidrug Resistant Neisseria gonorrhoea infection in a patient with vasculitic skin rash

**DOI:** 10.1590/0037-8682-0289-2023

**Published:** 2023-09-22

**Authors:** Kok Soon Lee, Masliza Zaid, Edmund Liang Chai Ong

**Affiliations:** 1 Ministry of Health, Johor Bahru, Malaysia.; 2 Hospital Sultanah Aminah, Johor Bahru, Malaysia.; 3 Newcastle University Medicine Malaysia, Johor Bahru, Malaysia & Newcastle UK.

**FIGURE 1:**
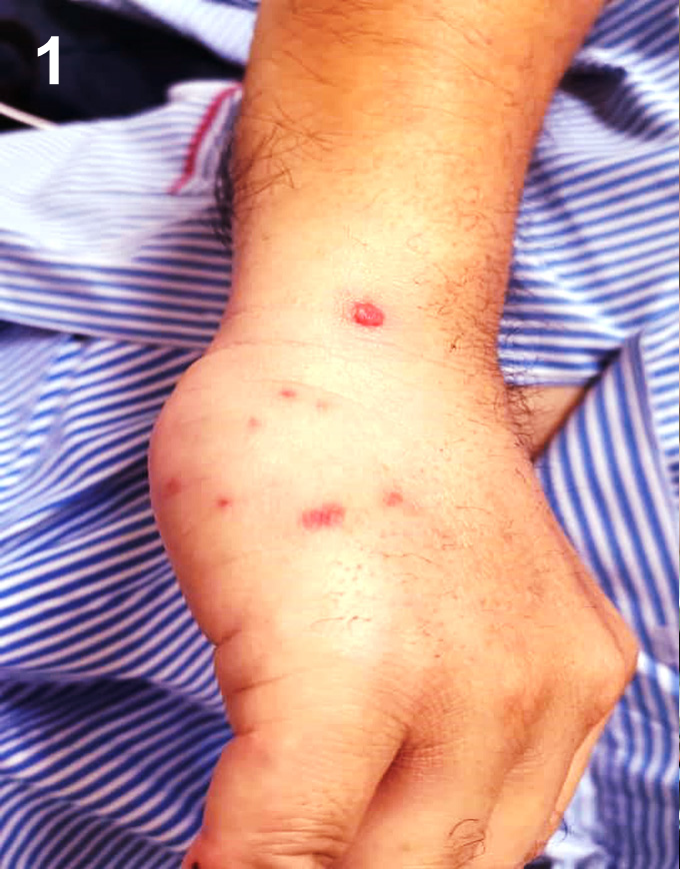
Vasculitic lesions over the right arm.

**FIGURE 2:**
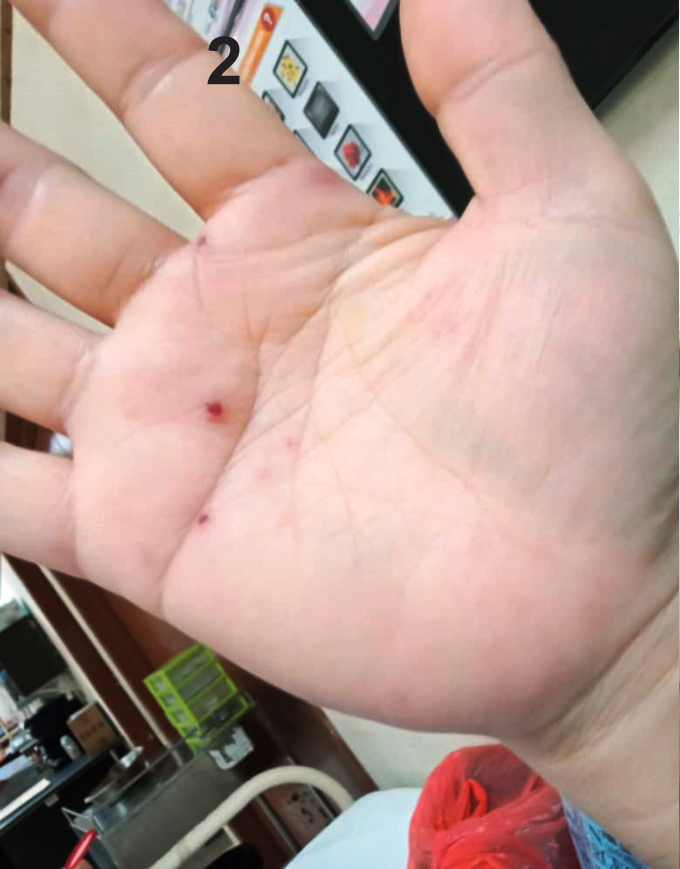
Vasculitic lesions over the palm.

**FIGURE 3:**
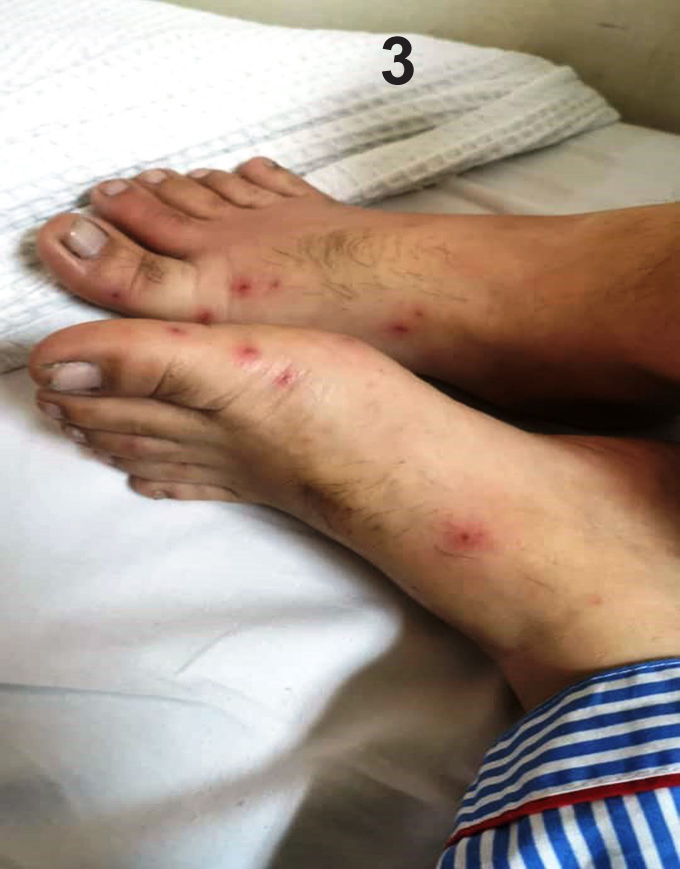
Vasculitic lesions over both feet.

A 48-year-old man was admitted with a 2-day history of a vasculitic skin rash on both his hands and feet. The rash first appeared on his hands before spreading to his lower limbs. Following the rash’s appearance, he developed a fever. His medical history included a metallic valve replacement in 2015 and long-term warfarin therapy. He was diagnosed with nasopharyngeal carcinoma in February 2021 and was awaiting chemotherapy. The rash was painful, non-blanching, multiple, raised, smooth, and erythematous (Figure 1, 2, and 3). A cardiovascular examination revealed no murmur, but a clear, crisp mitral prosthetic click was audible. The abdominal examination was unremarkable. Infective endocarditis was initially suspected, and treatment with penicillin and gentamicin was initiated. However, a trans-thoracic echocardiography showed no vegetation. Blood cultures subsequently grew Gram-negative bacilli identified as *Neisseria gonorrhoeae*, which was resistant to penicillin, tetracycline, and ciprofloxacin but sensitive to ceftriaxone. The treatment was switched to intravenous ceftriaxone in place of penicillin and gentamicin, which was continued for 2 weeks, resulting in complete resolution. Further investigations, including a transoesophageal echocardiography and a sexual health screen for HIV, hepatitis B and C, syphilis, and *Chlamydia trachomatis* IgM, were negative. The majority of gonococcal infections in men are asymptomatic^1^ ^,^ ^2^ and can lead to disseminated bacteraemia, as demonstrated in this case. Gonococcal vasculitic lesions can also present a diagnostic challenge, and there is increasing awareness of drug resistance^3^.
